# The role of plant polyploidy in the structure of plant-pollinator communities

**DOI:** 10.3389/fpls.2026.1676445

**Published:** 2026-02-10

**Authors:** Ido Zylberberg, Keren Halabi, Noa Ecker, Nathália Susin Streher, Tal Pupko, Tia-Lynn Ashman, Itay Mayrose

**Affiliations:** 1 School of Plant Sciences and Food Security, George S. Wise Faculty of Life Sciences, Tel Aviv University, Tel Aviv, Israel; 2 The Shmunis School of Biomedicine and Cancer Research, George S. Wise Faculty of Life Sciences, Tel Aviv University, Tel Aviv, Israel; 3 Department of Biological Sciences, University of Pittsburgh, Pittsburgh, PA, United States

**Keywords:** plant-pollinatorcommunities, extinction, visitation networks, plant-pollinator interactions, polyploidy

## Abstract

Polyploidization is a major macromutation, bearing notable genomic and ecological consequences. While the impact of polyploidy on plant abiotic niches is well studied, our understanding of its consequences on biotic interactions, and particularly pollination, is lacking and hardly includes its role in shaping plant-pollinator community structure. Here, we integrate hundreds of plant-pollinator networks, ploidy inferences, reproductive traits, and climatic attributes to ascertain whether a general pattern characterizes the link between polyploid frequency and network structure. We further examine whether environmental factors and plant traits known to be associated with polyploidy mediate this relationship. Our analysis reveals that an increased frequency of polyploid species within networks is positively associated with network nestedness while being negatively associated with modularity. Path analysis reveals that these associations are partially mediated via the frequency of self-compatible plants and by differences in flower shape. Despite these alterations in community structure, the heightened abundance of polyploids appears to have minimal impact on network connectance and resilience to extinction. Our findings indicate that unlike abiotic interactions, the relationships between polyploidy and biotic interactions are less predictable and reflect the combined contributions of phenotypic and environmental factors. However, we acknowledge that incomplete data sets limit a clear understanding of the causal relationship that may exist.

## Introduction

Polyploidy, the possession of more than two sets of chromosomes resulting from whole genome duplication (WGD), is a prominent evolutionary process in plants. Most plant species have experienced WGD at some point in their history (Cui et al., 2006; Jiao et al., 2011), and recent polyploidization events are estimated to have occurred in approximately 35% of extant flowering plant species (Wood et al., 2009; Halabi et al., 2023). Polyploidy has far reaching genomic consequences (Soltis and Soltis, 2000; Otto, 2007), contributing to the emergence of novel genetic variation that facilitates ecological diversification and rapid niche differentiation (Levin, 1983; Ramsey and Schemske, 2003; Martin and Husband, 2009; Van De Peer et al., 2017; Baniaga et al., 2020), and potentially affects community assembly and stability (Segraves, 2017). Nevertheless, our understanding of the ecological consequences of polyploidy remains limited (Ramsey and Ramsey, 2014; Segraves, 2017).

To date, research on the ecological effects of polyploidy has mostly centered on the abiotic niche (Brittingham et al., 2018; Baduel et al., 2018; Rice et al., 2019; Wei et al., 2019; Baniaga et al., 2020). Broad-scale comparative analyses have revealed, for example, that polyploids tend to exhibit elevated rates of climatic niche differentiation from their parental species compared to diploids (Baniaga et al., 2020), and that polyploids are more common in regions characterized by cold climates and lower species richness (Rice et al., 2019). Similar broad-scale analysis has yet to be performed on the association between polyploidy and biotic interactions (Segraves and Anneberg, 2016; Segraves, 2017; Gaynor et al., 2018; Streher et al., 2024; Anneberg et al., 2023) and consequently, it is yet to be evaluated whether polyploids contribute in distinctive ways to ecological communities.

Animal-mediated pollination is a biotic interaction essential for the successful reproduction of the majority of flowering plant species (Ollerton et al., 2011; Rodger et al., 2021), and has a significant impact on plant population dynamics (Ashman et al., 2004) and community structure (Sargent and Ackerly, 2008; Wei et al., 2021; Bascompte & Scheffer, 2023). Changes in temperature and precipitation regimes have been shown to alter pollinator activity and abundance (Doré et al., 2021) which can increase competition for pollinators among co-flowering plants (Mustajärvi et al., 2001; Lundgren et al., 2016) and potentially reduce the stability of plant–pollinator communities (Johnson et al., 2022). However, increased self-fertilization, associated with several plant traits (Rodger et al., 2021), could lessen these consequences.

Polyploidy has also been linked with several floral traits, including flower size, floral morphology, and self-compatibility (Ramsey and Schemske, 2003; Barringer, 2007; Porturas et al., 2019), which can influence pollinator interactions. However, the relationship between polyploidy and self-compatibility is complex and varies among self-compatibility systems. Broad-scale comparisons found no general association between polyploidy and self-compatibility/incompatibility status (Mable, 2004), whereas more focused studies have shown that particular forms of self-compatibility may facilitate polyploid establishment by mitigating the minority-cytotype disadvantage (Robertson et al., 2011; Duan et al., 2024). Polyploidization can also generate phenotypic changes relevant to pollinator use (e.g., via the gigas effect on flower size or shape), raising the possibility that polyploids sometimes occupy broader or altered pollination niches relative to diploids. However, patterns of ecological niche differentiation following polyploidization are not universal, with some studies showing that niche breadth can expand, contract, or remain conserved depending on lineage history (Padilla-García et al., 2023; Pittet et al., 2025). If polyploidy is associated with variation in pollination-niche breadth, then variation in the frequency of polyploids may help explain broad differences in community-level network structure.

Emerging polyploid lineages face an initial frequency disadvantage because crosses with the majority diploid cytotype often yield inviable seeds or low-fertility triploid offspring (Ramsey and Schemske, 2003; Köhler et al., 2010; Osterman et al., 2025). The success of a new polyploid individual in accessing mates of its own cytotype and overcoming this frequency disadvantage may be influenced by pollination, via two possible ecological trajectories. First, polyploidization can generate changes in floral morphology or phenology (e.g., the *gigas* effect; Ramsey and Schemske, 2003) that may modify pollinator visitation patterns and shift the pollination niche (Segraves and Anneberg, 2016; Casazza et al., 2017), although evidence that such changes alone create sufficient assortative mating for establishment is limited. On the other hand, narrowing the pollination niche of polyploids could be obtained via higher self-pollination rates, reducing reliance on pollinators (Rodger and Ellis, 2016). Under both alternatives, shifts in pollination niche could contribute to conditions that lessen competitive or mating disadvantages experienced by minority cytotypes and thereby influence the potential for polyploids to persist alongside diploids. Certainly, understanding the dynamics of pollination niche changes is therefore relevant for elucidating factors that govern the establishment and persistence of polyploid lineages in sympatry with diploids (Casazza et al., 2017; Segraves, 2017) and ultimately how these lineages fit within their communities. While species-level processes determine the establishment and persistence of polyploid lineages, it remains unclear whether variation in polyploid frequency is associated with broader community-level patterns. Here, we address this gap by examining how polyploid frequency relates to the structure of plant–pollinator networks across many communities.

**Figure 1:**
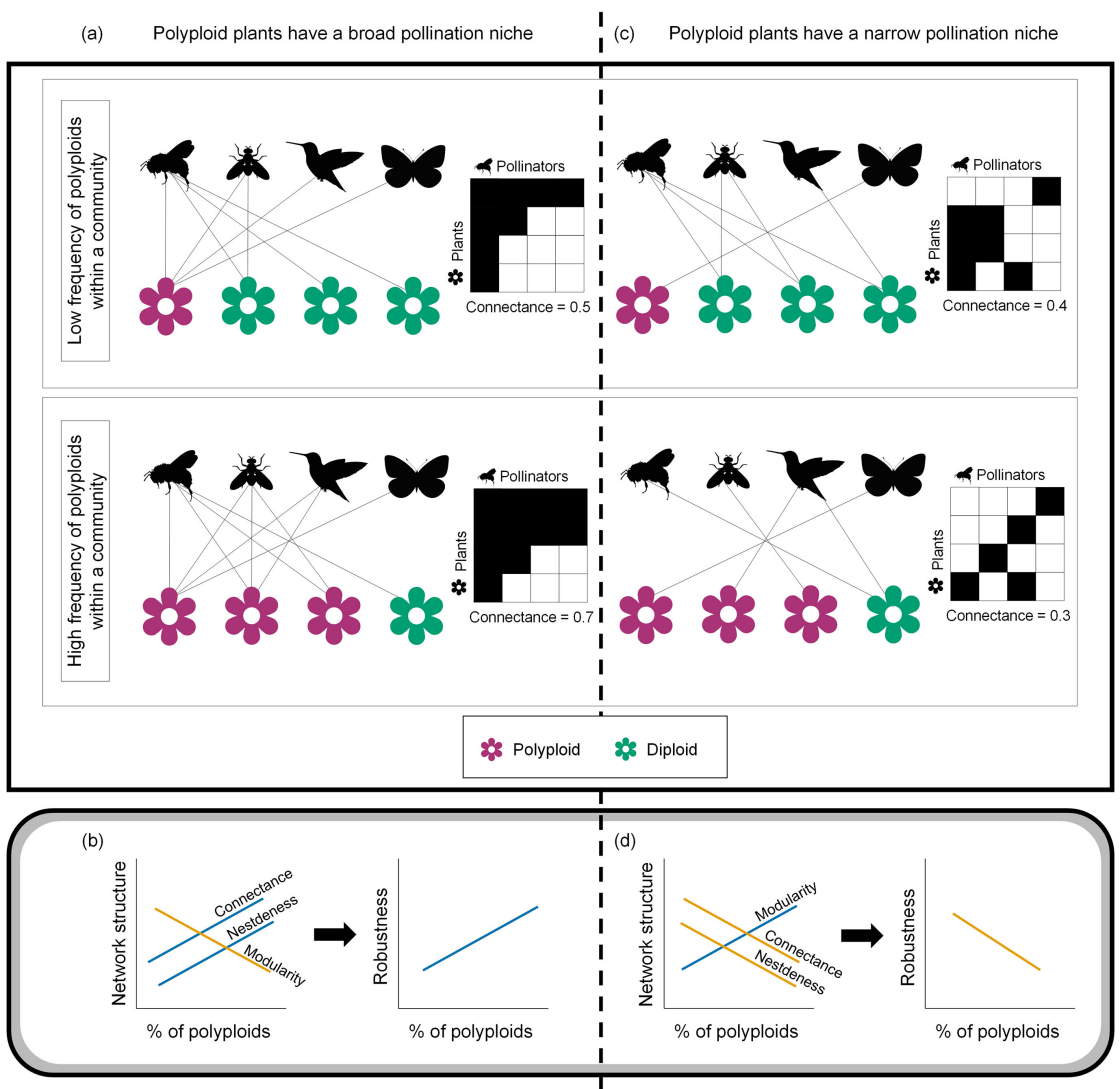
Examples of how the frequency of polyploid plants in a community can impact network structure under different pollination niche scenarios. In scenario **(a)**, polyploids have a broader pollination niche relative to diploids, thus **(b)** the increase of polyploid frequency in a community is expected to increase the network indices connectance and nestedness that can confer higher robustness to the community. In scenario **(c)**, polyploids have a narrower pollination niche relative to diploids, thus **(d)** the increase of polyploid frequency is expected to increase modularity but reduce connectance which could cascade to a less robust community. Polyploid plants are represented as pink flowers and diploid plants as green flowers. In panels **(b,d)**, orange lines correspond to negative relations and blue to positive ones.

A broad characterization of the role of polyploids within their communities can be obtained from plant-pollinator interaction networks (Vazquez et al., 2012). Associations between polyploidy and network structure may arise either directly or indirectly via floral traits that covary with polyploid presence. Several floral traits, including flower shape and size, are known to influence pollinator visitation and pollination-niche structure (Dafni and Kevan, 1997; Lázaro et al., 2020; Delgado et al., 2023), and functional traits are major drivers of the structural properties of plant-pollinator networks (Olesen et al., 2007; Martín González et al., 2012). Because polyploidy mediates a suite of floral differences and potentially niche breadth, a higher presence of polyploid species is expected to be reflected in overall network structure, as illustrated in **Figure 1**. Notably, because trait–pollinator relationships vary geographically and among lineages (Ollerton et al., 2009; de los Santos-Gómez et al., 2022; Rosas-Guerrero et al., 2014) it is necessary to examine how polyploid frequency, associated floral traits, and plant–pollinator interactions are jointly related across many communities to gain broad-scale insight.

Network-wide indices, including connectance, nestedness, and modularity, summarize different structural aspects of mutualistic interaction networks and have been proposed to relate to community robustness, defined as the ability of the network to withstand species loss (Duan et al., 2023). Connectance reflects the proportion of realized interactions in a network and increases with the diversity of interacting species. Nestedness measures the degree to which the interaction partners of specialists form subsets of those of generalists (Bascompte et al., 2003). High nestedness has been suggested to indicate reduced competition, potentially enhancing the resilience of the community to disturbances (Bastolla et al., 2009; Fortuna et al., 2010), although such effects depend on the nature of plant phenotypes within the community (e.g., co-flowering plants sharing a pollinator could compete with one another). Modularity quantifies the tendency of interactions to form tightly interconnected subgroups (Newman and Girvan, 2004; Guimerà and Amaral, 2005). Higher modularity can localize the effects of disturbances within modules but may also reduce adaptability if modules are too isolated (Xiang et al., 2023). Here, we provide a comprehensive assessment of the relationship between the frequency of polyploids within their communities and the structure of plant-pollinator networks. We hypothesize that if polyploids, in general, have broader pollination niches than diploids (**Figure 1a**), then increased presence of polyploids would be associated with enhanced aspects of network structure, such as connectance, that promote stability of the community (Okuyama and Holland, 2008; Martín González et al., 2010; Thébault and Fontaine, 2010; Johnson and Ashman, 2019). This could also lead to increased nestedness and decreased modularity, particularly in larger networks (**Figure 1b**) (Fortuna et al., 2010; Landi et al., 2018). On the other hand, a narrowed polyploid pollination niche (**Figure 1c**) would correspond to polyploids having more peripheral roles within their communities, increased modularity, and reduced connectance and robustness in the face of extinction (**Figure 1d**) (Thébault and Fontaine, 2010; Theodoridis et al., 2013; Baduel et al., 2018; Spoelhof et al., 2020). Furthermore, we hypothesize that the effect of polyploidy on network structure could be mediated through associated functional traits. Specifically, higher frequencies of self-compatible polyploids may be linked to increased specialization of the network, reflected in higher modularity and lower nestedness and connectance. On the other hand, increased frequency of polyploids exhibiting flower shapes that are permissible to pollinators would contribute to generalization aspects of the associated networks, namely, reducing modularity and increasing nestedness and connectance.

To examine how polyploid frequency is related to variation in community structure, we assembled an extensive dataset of 325 plant-pollinator networks, spanning 5,054 unique pollinators and 1,527 plant species of which 1,005 have ploidy classification. These were supplemented with environmental data as well as data on mating system and flower shape. In our analysis we chose to characterize flower shape as ‘restrictive’ or ‘not restrictive’ because these directly reflects the potential for pollinators to access floral rewards and mediate pollination (i.e., it is a function of a variety of floral morphological traits; Burns et al., 2019). Mating system (categorized here as self-compatible or self-incompatible) facilitates polyploid persistence and self-compatible plants were shown to have narrower pollination niche (Streher et al., 2024). We further included environmental variables evaluated in previous work as potential drivers of polyploidy (Rice et al., 2019) or plant–pollinator network structure (Liu et al., 2021): temperature seasonality (BIO4), mean temperature of the warmest quarter (BIO10), precipitation seasonality (BIO15), and precipitation of the warmest quarter (BIO18). With this data, we specifically test (1) whether higher polyploid frequency within communities is associated with predictable changes in plant-pollinator network structure. In particular, we evaluate whether increased polyploid abundance is consistent with a broadened pollination niche, as manifested by higher nestedness, higher connectance, and lower modularity, or alternatively with a narrowed niche, which would manifest as the opposite pattern (**Figure 1**). We then assess (2) whether these associations, if they exist, are related to associations with environmental factors and reproductive traits linked to polyploidy, and (3) whether variation in polyploid frequency impacts the network’s resilience to extinctions.

## Materials and methods

### Assembly of plant-pollinator networks and trait data

A plant-pollinator community can be represented by a connectivity matrix depicting the presence/absence of visits by pollinator taxa to a plant species (denoted as a binary network) or the frequency of pollinator visits (denoted as a weighted network). Here, we refer to plant-pollinator interaction networks although we acknowledge some published networks in our data set may have recorded flower visitation but not confirmed effective pollination by the visitors per se. To examine the effect of polyploidy on interactions of plants with pollinators, we collected 404 weighted networks from three online resources: Interaction Web Database (IWDB) (http://www.ecologia.ib.usp.br/iwdb/), Mangal DB (Poisot et al., 2016), and Web of life (Bascompte, 2009), with data accessed during January 2023. These were supplemented with a literature search that retrieved 335 additional unique networks (Aizen et al., 2008; Trøjelsgaard et al., 2019; Schwarz et al., 2020). In total, 739 weighted networks were collected. The geographic locations of the plant-pollinator community from which the networks were obtained were extracted from the three online databases or, if not available, via manual data mining from the source manuscripts. We then mapped the coordinates (longitude, latitude) to climatic data in the years for 1970–2000 using the R package raster (Hijmans, 2023a) and WorldClim version 2.1 (Fick and Hijmans, 2017). The WorldClim Global Climate Data consists of 19 temperature and precipitation characteristics (BIO1 to BIO19) that were developed from climate data during 1970–2000 at a ten arc-minute resolution. Four climatic factors were used in our analysis: temperature seasonality (BIO4), precipitation seasonality (BIO15), mean temperature of warmest quarter (BIO10), and precipitation of warmest quarter (BIO18). We anticipated that two of these could act as drivers of plant-pollinator interaction dynamics: BIO15 has been directly associated with increased modularity and decreased robustness (Liu et al., 2021); and BIO10 was shown to be strongly associated with polyploid distribution across the globe (Rice et al., 2019) and could consequently act as a driver of changes in plant-pollinator interactions. BIO4 and BIO18 were chosen as complementary to BIO15 and BIO10. The collected networks and their respective metadata are available in **Supplementary Files S1, S2 and S3**.

To enable integration of data from multiple sources and to assess the taxonomic resolution of the networks, name resolution was applied for both plants and pollinators appearing in the networks. Name resolution is a procedure that maps the original names recorded in an empirical dataset to their currently accepted names, while correcting for possible misspellings. Name resolution was applied using the Taxonome tool (Kluyver and Osborne, 2013) against the World Flora Online for plant names (Borsch et al., 2020), and against data retrieved from the Integrated Taxonomic Information System (ITIS; www.itis.gov) for pollinator names. This resulted in 79% (4,261 records) of the plant names and 21% (2,885 records) of the pollinator names resolved at the species level. Notably, most pollinator names were documented at the species level but were resolved at genus level due to name ambiguity (e.g., *Pollenia* sp1 subsp. m_pl_006), with only 10% of the unresolved names consisting of a single word. Plant classification as either polyploid or diploid was obtained from PloiDB (Halabi et al., 2023) based on resolved names (or original name if unresolved). Here, we used classifications devised from inferences at the genus-level, which were found to produce the highest agreement with data reported in the literature, and mostly refer to neo- and meso-polyploids (Halabi et al., 2023).

To quantify the mediated effect of polyploidy through associated plant traits, we assembled a database of plant reproductive traits known to be impacted by polyploidy (mating system and flower restrictiveness; Vamosi et al., 2007; Streher et al., 2024). Mating system data (self-compatible or self-incompatible) was collected from two main compilations (Grossenbacher et al., 2016; Zenil-Ferguson et al., 2019) and further supplemented using a literature search. Additionally, following previous studies (Burns et al., 2019; Lanuza et al., 2023), we categorized plant species into those with flower shapes that restrict pollinator access to reproductive parts or rewards (e.g., corolla tubes, nectar spurs, flag/keel, poricidal anthers, etc.) and those with minimal or no morphological restrictions (e.g., open flowers, fully exposed reproductive organs, etc.). Floral restrictiveness assignments were scored using a standardized four-level morphological framework (unrestrictive, low restriction, moderate restriction, high restriction), detailed in **Supplementary Note S1**. Each species was assigned a category based on floral morphology, with an accompanying justification and source (**Supplementary File S4**). For the statistical analyses, flower restrictiveness was treated as a binary variable by grouping ‘unrestrictive’ and ‘low restriction’ together and ‘moderate’ and ‘high restriction’ together. This binning provides a consistent and interpretable trait representation across models while retaining the broader biological distinction between accessible versus more restrictive floral forms.

To ensure sufficient information for network-level analyses, we retained only weighted networks that met a minimum set of criteria: each network had to contain at least six pollinator taxa, at least six plant species with assigned ploidy information, and ploidy classifications available for at least 33% of the plant species. We additionally excluded networks for which climatic data were unavailable, resulting in 325 networks. For the path analysis we additionally filtered networks with no information on the frequency of restrictive flowers (hereafter %Restrictive) or of self-compatible plants (hereafter %SC), resulting in 313 networks.

### Modeling the link of polyploid frequency with community structure

#### Measures of community structure

To test whether polyploid frequency within a community is associated with network architecture, we computed four network indices across the set of weighted networks collected here. These indices capture key structural aspects of the interaction network: (1) Connectance, defined as the proportion of realized interactions out of all possible interactions across species within the network. It represents the actual connections formed compared to the total potential connections in the network. To compute this measure, all networks were treated as binarized; (2) Nestedness, defined as the degree to which the network consists of nested substructures, in which specialist members primarily interact with members that generalists also interact with, and computed as weighted NODF (nested overlap and decreasing fill (Almeida-Neto et al., 2008)) (3) Modularity, defined as the tendency of a network to be organized in modules, such that members within a module mostly interact among themselves and little with members from other modules, and computed using the Q index (Newman and Girvan, 2004). The modules within each network were detected using the optimization algorithm of Beckett (2016); (4) Robustness, defined as the ability of a community to endure extinctions of its members, and computed as the area under the extinction curve under random extinction of plant species, following Burgos et al. (2007). Following previous studies (Escobedo-Kenefic et al., 2020; Stein et al., 2021; Vollstädt et al., 2022; Herrmann et al., 2023), we delta-transformed all indices relative to a null distribution (i.e., subtracted the mean across null simulated networks from the raw values). The null simulations were conducted while preserving the network size using the algorithm of Patefield (1981). The null simulations and computation of all indices were obtained using the bipartite R package (Dormann et al., 2009).

#### Statistical analysis

**Figure 2:**
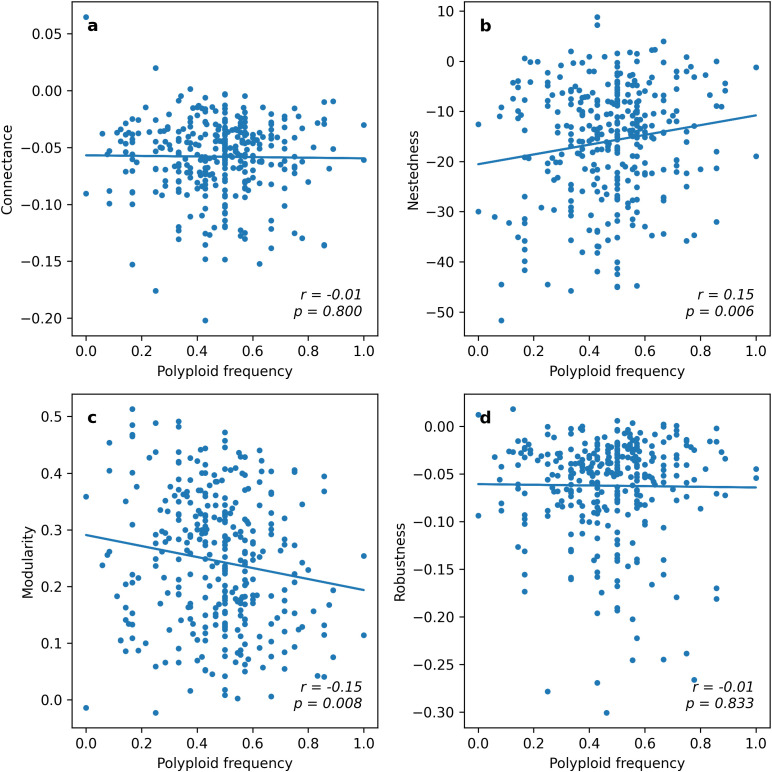
The distribution of standardized plant-pollinator network metrics plotted against increasing frequencies of polyploid taxa in the networks. Metrics of connectance **(a)**, nestedness **(b)**, modularity **(c)**, and robustness **(d)** are shown across the 325 weighted networks included in the analysis. Pearson correlation coefficients (*r*) and their significance levels (*p*) are shown at the bottom right-hand side of each plot. .

The polyploid frequency within a network (hereafter, %PP) is defined here as the proportion of plant species classified as polyploids out of all the plant species within the network for which a ploidy classification was available. We first conducted a regression analysis to examine the effect of %PP, as well as that of other reproductive traits and environmental factors, on the computed network indices (**Figure 2**). This was carried out independently for each of the four network indices (connectance, nestedness, modularity, and robustness). Specifically, we initially examined the following predictors corresponding to reproductive trait frequencies within the networks: frequency of restrictive flowers (%Restrictive) and of self-compatible plants (%SC). Similar to %PP, reproductive trait frequencies were computed relative to the number of plant species within the network for which a trait value was available. As such, each frequency value possesses a varying degree of missing data. The four environmental factors (BIO4, BIO10 – corresponding to temperature seasonality and mean value at the warmest quarter, and BIO15, BIO18 – corresponding to similar measures of precipitation) were also included. To overcome skewness, and following a previous study (Poppenwimer et al., 2023), the precipitation variables were log transformed, as was network size (e.g., as in Liu et al., 2021).

To assess the association of each variable on each network-based index, univariate linear models were fitted to the different response indices (connectance, nestedness, modularity, robustness), each time with a different predictor. Because trait information varied in availability across networks, the univariate regressions were performed using all networks from the filtered dataset (n = 325) for which the focal predictor variable was available. Thus, the sample size differed among predictors (i.e., 325 networks for %PP and all climatic variables, 101 for %SC, and 316 for %Restrictive). This approach maximizes statistical power for each predictor independently and avoids discarding networks unnecessarily. To account for spatial dependencies among samples, we tested the residuals of each model for spatial autocorrelation using the R package terra (Hijmans, 2023b). A significant positive spatial autocorrelation was detected for all models examined, and we thus fitted additional simultaneous autoregressive (SAR) models (Kissling and Carl, 2008) under the same settings using the R package spdep (Bivand, 2022). The results for the SAR models can be found in **Supplementary Table S1**.

**Figure 3:**
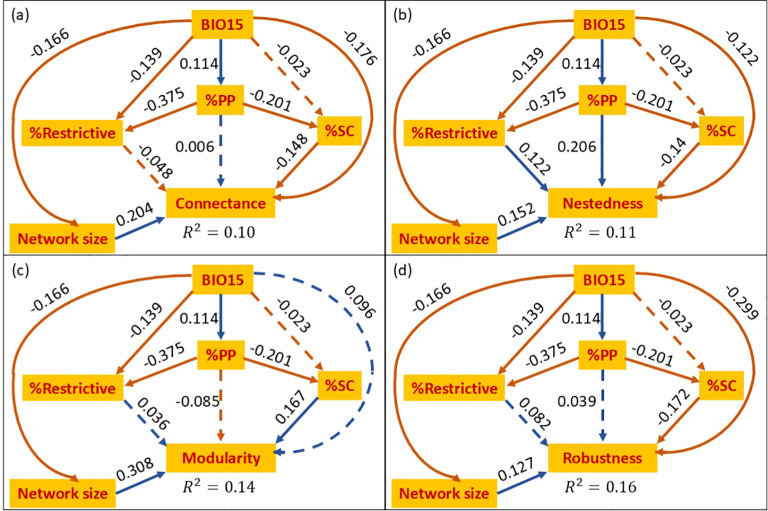
Path diagrams of the four examined network metrics: connectance **(a)**, nestedness **(b)**, modularity **(c)** and robustness **(d)**. BIO15 is precipitation seasonality, %PP corresponds to the frequency of polyploids within each network, and %SC and %Restrictive correspond to the frequencies of self-compatible plants and those with respective flower shape, respectively. Arrows denote the direction of modeled relationships and are not intended to reflect biological causation. Full lines correspond to paths with significant contribution and dashed lines correspond to paths with a non- significant contribution. Orange lines correspond to negative coefficients and blue to positive ones. The standardized coefficients are shown next to each edge in the diagram. The $R^{2}$ is shown next to each network index. In all panels, the results of the $\;\chi^{2}$ test for model adequacy were non-significant (*p* = 0.161), indicating that the model is adequate.

To evaluate whether associations between polyploid frequency and network structure could arise directly or via plant traits, we conducted a path analysis using the R package piecewiseSEM (Lefcheck, 2016). For each network index, we specified an identical structural equational model informed by theoretical expectations (**Figure 3**). In these models, %PP was assumed to have a direct association with the examined network index, and indirect paths through %SC and %Restrictive. Additionally, all plant factors (%PP, %SC and %Restrictive) and the examined network index were assumed to be affected by environmental conditions. To avoid using excessively complicated models, in this analysis we included a single environmental variable (BIO15: precipitation seasonality), which had the strongest overall contribution according to the univariate regression analyses. Additionally, we included network size in the diagram to account for variability in network indices related to network size and accommodated for environmental influence on network size as an edge in the diagram. We note that the directional arrows in the SEM represent conditional dependencies required by the statistical model and do not imply biological causation. During fitting, we verified that no additional edges should be added to the diagram using d-separation tests (Shipley, 2013). The path analysis requires all predictors simultaneously and therefore was fitted using the 313 networks that contained sufficient information on ploidy, the central variable of interest in this study, and at least some data for either %SC or %Restrictive. Applying a more restrictive three-way filter to retain only networks with sufficient information for all three plant traits (%PP, %SC, and %Restrictive) reduced the dataset to 101 networks, eliminating statistical power. We report the results of this highly restricted analysis in **Supplementary Figure S2**. To further assess the robustness of the path analysis, we explored several variants of the initial model: (1) using BIO10 (mean temperature of warmest quarter; the most influential temperature-based variable in the univariate analysis) as an alternative environmental factor (**Supplementary Figure S4**); (2) including both BIO10 and BIO15 as environmental predictors (**Supplementary Figure S5**); (3) excluding the %Restrictive factor from the diagram (**Supplementary Figure S6**); (4) excluding the %SC factor from the diagram (**Supplementary Figure S6**).

## Results

### Network data assembly

We assembled an extensive dataset of plant-pollinator interaction networks and supplemented these with ploidy estimates. Initially, a total of 725 weighted networks were retrieved. Following data filtration to ensure the analyses of networks of sufficient quality, our main dataset consisted of 325 networks that were globally distributed (**Supplementary Figure S1**), but somewhat biased toward temperate northern localities, reflecting the uneven geographic coverage of available studies (Vizentin-Bugoni et al., 2018). These 325 networks contained, on average, 15.7 plant species and 44.3 pollinator taxa, overall encompassing 5,099 plant vertices and 14,403 pollinator vertices and representing 1,527 unique plant species across 122 families and 5,054 unique pollinator names. We obtained ploidy classifications for 1,005 plant species, flower restrictiveness scores for 1,012 species, and mating system information for 318 species. The distributions of these plant traits across the analyzed networks are shown in **Supplementary Figure S2**, and species-level trait information is provided in **Supplementary File S4**.

### The association of polyploid frequency with community structure

To quantify the association of polyploid frequency as well as other potential factors on network structure, we first conducted a series of univariate regression analyses. Each predictor was analyzed using all networks for which sufficient data for that predictor were available, and the analysis was carried out independently for the four network indices that characterize different aspects of network structure: connectance, nestedness, modularity, and robustness. These analyses showed that frequency of polyploids within networks (%PP; *n* = 325 networks) was positively associated with nestedness (*r* = 0.151, *p* = 0.006), and negatively with modularity (*r* = -0.147, *p* = 0.008), while the associations with connectance and robustness were nearly zero and statistically non-significant (**Figure 2**; **Table 1**). The non-significant impact of %PP on robustness was further supported by additional extinction simulations (varying by which type of plants went extinct first), which indicated that the resilience of networks is largely unaffected by whether initial extinctions target diploids or polyploids (**Supplementary Note S3**). The two floral traits showed weaker effects overall. Floral restrictiveness (%Restrictive, *n* = 316 networks) showed a positive association with robustness (*r* = 0.135, *p* = 0.016), a marginally positive association with modularity (*r* = 0.1, *p* = 0.074) and no significant associations with connectance or nestedness. For the frequency of self-compatible plants within networks (%SC, *n* = 101 networks), none of the associations with the four network indices were statistically significant. These results should be interpreted cautiously, as mating-system information is available for relatively few species in many networks, severely reducing statistical power in these analyses.

**Table 1:** Results of the univariate regression analysis.

Predictor	N networks	Connectance		Nestedness		Modularity		Robustness	
		R ( β)	p	R ( β)	p	R ( β)	p	R ( β)	p
%PP^a^	325	-0.014 (-0.253)	0.8	**0.151 (2.748)**	**0.006**	**-0.147 (-2.674)**	**0.008**	-0.012 (-0.211)	0.832
%SC^b^	101	-0.044 (-0.438)	0.658	0.015 (0.149)	0.881	0.144 (1.445)	0.146	-0.044 (-0.438)	0.659
%Restrictive^c^	316	-0.014 (-0.256)	0.797	0.077 (1.377)	0.168	0.100 (1.783)	0.074	**0.135 (2.405)**	**0.016**
BIO4^d^	325	**-0.144 (-2.622)**	**0.009**	-0.07 (-1.268)	0.204	0.05 (0.893)	0.37	**-0.295 (-5.546)**	**0**
BIO10^e^	325	0.004 (0.068)	0.946	0.023 (0.408)	0.683	**0.109 (1.966)**	**0.049**	-0.018 (-0.329)	0.742
BIO15^f^	325	**-0.188 (-3.434)**	**0.001**	**-0.148 (-2.687)**	**0.007**	0.027 (0.486)	0.626	**-0.323 (-6.127)**	**0**
BIO18^g^	325	0.075 (1.352)	0.176	0.01 (0.188)	0.851	-0.004 (-0.068)	0.946	**0.149 (2.715)**	**0.007**
Network size	325	**0.224 (4.139)**	**0**	**0.153 (2.786)**	**0.005**	**0.304 (5.741)**	**0**	**0.178 (3.257)**	**0.001**

^a^Frequency of polyploid species in the network; ^b^Frequency of self-compatible species; ^c^Frequency of species with restrictive floral morphology; ^d^Temperature seasonality; ^e^Mean temperature of warmest quarter; ^f^Precipitation seasonality; ^g^Precipitation of warmest quarter.

For each network-based index, the Pearson correlation coefficient ( R) and its standardized value ( β) are displayed, along with the p-value of the respective regression model. Significant associations are bolded.

Among the environmental factors examined (*n* = 325 networks), networks with lower connectance and robustness were generally located in regions with high temperature or precipitation seasonality (BIO4 and BIO15, respectively; **Table 1**). Lower robustness was also associated with regions having dry summers (lower values of BIO18). Environmental associations with nestedness and modularity were generally less prominent, although nestedness was significantly lower in regions with high precipitation seasonality. The direction of all associations remained unchanged upon replacing linear models with spatial autoregressive models (**Supplementary Table S1**), although several effects became non-significant, suggesting that spatial structure may be stronger than the predictive power in some cases.

### The direct and indirect association of polyploid frequency with community structure

While the univariate regressions identified associations between individual predictors and network structure, they do not reveal the relative contributions of predictors or distinguish direct from indirect pathways. To disentangle these effects and evaluate whether associations with polyploid frequency could be mediated by reproductive traits, we conducted a path analysis. Because path analysis requires all predictors simultaneously, it was fitted using the 313 networks that contained information on ploidy, the central variable of interest in this study, and at least some data for %SC and %Restrictive. We acknowledge that not all networks contained complete trait information, and therefore the path analysis should be interpreted with appropriate caution.

Using this dataset, the results of the path analysis broadly aligned in direction with the univariate regressions and clarified the relative magnitudes of the modeled associations among predictors (**Figure 3**). The direct association of polyploid frequency was most noticeable with that of nestedness (standardized coefficient = 0.206), a weaker direct negative association with modularity (-0.085), and weak associations with connectance (0.006) and robustness (0.039). Among the reproductive traits, the standardized coefficients for %SC were relatively large within the model structure (0.14-0.17 in absolute values), although these associations should be interpreted cautiously given that mating-system information was incomplete for many networks. Floral restrictiveness (%Restrictive) had weaker associations overall but included a significant positive association with nestedness (0.12).

Polyploid frequency was negatively associated with both %SC (–0.20) and %Restrictive (–0.375), indicating that polyploid-rich communities tend to contain fewer self-compatible and fewer restrictive-flowered species. Decomposing the total association of %PP into the direct and indirect components (**Table 2**), revealed that their relative magnitudes varied across network indices. For nestedness, the direct association of %PP dominated its combined indirect pathways via both %SC and %Restrictive (direct effect of 0.206 versus combined indirect effect of -0.018). This was not the case for the other indices where the direct pathway of %PP was weaker (connectance) or similar (robustness and modularity) than the indirect components. The analysis further indicated that precipitation seasonality (BIO15) had direct negative associations with both connectance and robustness, consistent with the patterns observed in the univariate regressions.

**Table 2:** Direct, indirect and total effects of polyploid frequency (%PP) on the four examined plant-pollinator network indices.

Response	Direct/indirect	Effect^a^
Connectance	Direct effect of %PP	0.006
	Indirect via %SC	0.030
	Indirect via %Restrictive	0.018
	Total effect of %PP	0.054
Nestedness	Direct effect of %PP	0.206
	Indirect via %SC	0.028
	Indirect via %Restrictive	-0.046
	Total effect of %PP	0.188
Modularity	Direct effect of %PP	-0.085
	Indirect via %SC	-0.034
	Indirect via %Restrictive	-0.013
	Total effect of %PP	-0.132
Robustness	Direct effect of %PP	0.039
	Indirect via %SC	0.035
	Indirect via %Restrictive	-0.031
	Total effect of %PP	0.043

a Direct paths are the standardized coefficients ( β); indirect paths are the product of the standardized coefficients across their respective edges; total effect is the sum of all effects.

The total effects of indirect paths via frequency of self-compatible (%SC) and frequency of restrictive flowers (%Restrictive) computed in the path analysis (**Figure 3**) are shown.

To assess the robustness of the path analysis, we also fitted this model to a more restricted dataset containing only networks with complete information for all three plant traits (%PP, %SC, and %Restrictive; 101 networks). As expected given the small sample size, none of the paths of the three plant traits with the network indices were statistically significant. In addition, we explored several variants of the initial model: (1) substituting BIO10 (mean temperature of the warmest quarter) for BIO15; (2) including both BIO10 and BIO15 as environmental predictors; (3) excluding %Restrictive; and (4) excluding %SC. These alternative specifications yielded qualitatively similar conclusions regarding the direction and relative magnitude of associations. Full results of all these analyses are provided in **Supplementary Figures 4–6**.

## Discussion

Our analysis of hundreds of plant-pollinator networks demonstrated that polyploidy, a profound and widespread evolutionary process in plants, is associated with key aspects of plant-pollinator network structure. These associations were at times subtle, and the path analysis indicated that the direct associations between polyploid frequency and network structure were generally stronger than the indirect contributions of polyploid frequency mediated through variation in the frequencies of self-compatible plants and species with restrictive flowers. Specifically, we found that networks with higher polyploid frequency tended to exhibit more nested and less modular structures. These patterns, however, did not propagate into increased connectance or enhanced robustness to extinction. Because our analyses are based on observational data, they cannot resolve the direction of causality. Nevertheless, the consistent covariation between polyploid frequency and network architecture provides broad evidence that genome duplication is linked to community-level interaction structure, motivating testable hypotheses about the ecological and evolutionary processes linking polyploidy and plant–pollinator communities.

The observed association between polyploid frequency and plant–pollinator network structure could arise through multiple non-exclusive causal pathways. One possibility is that polyploidy influences plant–pollinator interactions through changes in plant traits or reproductive strategies, which in turn reshape network architecture. Alternatively, properties of plant–pollinator networks may affect the establishment, persistence, or relative abundance of polyploids within communities, such that polyploids are more likely to accumulate in communities characterized by particular interaction structures. Below, we discuss how each of these pathways could give rise to the observed increase in nestedness and decrease in modularity in polyploid-rich networks.

Under the first scenario, polyploidy may influence plant–pollinator network structure by altering plant traits or reproductive strategies that affect pollinator interactions. Polyploidization is often associated with phenotypic changes relevant to pollination, including shifts in floral morphology, size, phenology, or reward presentation (Ramsey and Schemske, 2003; Porturas et al., 2019). Such changes could broaden the range of pollinators that interact with polyploid plants, potentially increasing overlap in pollinator use among plant species and contributing to more nested and less modular network structures. For example, flower size has been hypothesized to be larger in polyploids, attributed to the gigas effect (Ramsey and Schemske, 2003), and analyses of plant–pollinator interaction networks have shown that larger flowers are associated with broader pollination niches (Lázaro et al., 2020; Xiang et al., 2023). In this context, the observed reduction in modularity in polyploid-rich networks can be interpreted as reflecting reduced specialization and increased sharing of pollinators among plant species.

Alternatively, the observed associations may arise because properties of plant–pollinator networks influence the establishment, persistence, or relative abundance of polyploids within communities. Both theoretical and empirical studies indicate that pollination environments can shape the success of newly arisen polyploids, for example by mediating access to compatible mates or influencing the evolution of reproductive strategies such as self-compatibility (Husband, 2000; Oswald and Nuismer, 2011; Osterman et al., 2025). Under this scenario, polyploids may be more likely to persist in communities characterized by generalized interaction structures, such as highly nested networks, where pollinator sharing is extensive and mating opportunities may be less constrained. From this perspective, the association between higher polyploid frequency and reduced modularity does not necessarily imply that polyploidy alters network structure but rather features of interaction networks that facilitate the accumulation of polyploids within plant communities.

Regardless of the direction of causality, the combination of increased nestedness and reduced modularity observed in polyploid-rich networks is consistent with a shift toward more generalized interaction structures at the community level. Specifically, higher polyploid frequency within networks is associated with more hierarchical substructures, in which plants interact with subsets of the pollinators used by more generalist species, rather than with modular substructures in which interactions are compartmentalized among distinct plant–pollinator groups (**Figure 1**). This coupling of increased nestedness and decreased modularity has been shown to be prevalent in large and highly connected networks (Fortuna et al., 2010), and our analysis shows that this pattern persists after accounting for network size. Although increased nestedness has been linked with greater robustness to species extinction in mutualistic networks (Duan et al., 2023), we found no strong association between polyploid frequency and robustness. This may reflect the net outcome of multiple, potentially opposing effects on network resilience that are not captured by nestedness alone (Gómez et al., 2023).

At the community level, polyploid-rich networks tended to occur in plant communities with lower frequencies of self-compatible species and of species with restrictive floral morphologies, both of which are traits known to influence plant–pollinator interactions. These patterns suggest that differences in the composition of reproductive traits among communities may contribute to variation in network structure. We note, however, that mating-system information was unevenly available across networks, and therefore inferences regarding the role of self-compatibility should be interpreted with caution. In addition, floral traits beyond those considered here, such as flower size, phenology, or reward characteristics, have been shown to influence pollinator accessibility and interaction patterns (Stebbins, 1970; Bradshaw and Schemske, 2003; Faegri and van der Pijl, 2013; Cortés-Flores et al., 2017; Lázaro et al., 2020), and some of these traits may also covary with polyploidy. Thus, while trait composition likely contributes to the observed associations between polyploid frequency and network structure, no single trait axis alone appears sufficient to explain the community-level patterns detected across networks.

Beyond plant traits, environmental context was also associated with variation in plant–pollinator network structure and polyploid frequency. In particular, precipitation seasonality was negatively associated with connectance, nestedness, and robustness, and positively associated with modularity, consistent with previous findings at smaller spatial scales for robustness and modularity (Liu et al., 2021). We further found that precipitation seasonality was positively associated with polyploid frequency, suggesting that animal-pollinated plant communities in regions experiencing greater seasonal variation in precipitation tend to be more polyploid-rich. Together, these patterns suggest that climatic conditions form an important environmental context in which associations between polyploid frequency and plant–pollinator network structure are observed.

The analyses conducted here were performed at the network level, testing whether variation in the frequency of polyploids across communities is associated with differences in the structural characteristics of plant–pollinator networks. An alternative approach would be to focus on the species level, in which each species within a network acts as a data point, potentially allowing a more explicit examination of the role of individual polyploid species within communities. However, comparative species-level analyses across many networks are particularly sensitive to methodological heterogeneity, including differences in sampling design (e.g., visit-based versus pollen-based approaches; De Manincor et al., 2020), taxonomic resolution of the identified taxa, and temporal coverage. Such sources of heterogeneity can substantially affect species-level metrics; for example, a plant species whose pollinators were identified only at the genus level in one network may appear more specialized than in another network whose pollinators were documented at the species level. Temporal variation among networks sampled at the same location but in different years can further contribute to inconsistencies (Burkle and Alarcón, 2011; Chacoff et al., 2018). While such biases can also impact network-level analyses, their effects are generally more pronounced in species-level analyses, where each network contributes a different number of observations. Indeed, network-level indices were shown to be less sensitive to sampling methods compared to species-level ones (Gibson et al., 2011). Focused, taxon-specific studies (e.g., Streher et al., 2024) could provide an important complement to the network-level approach taken here but are less suited to addressing community-wide consequences of polyploidy across diverse systems.

Overall, our results indicate that variation in plant-pollinator community structure is associated with the relative abundance of polyploids within communities. Across a broad set of networks, polyploid-rich communities tended to exhibit higher nestedness and lower modularity, suggesting systematic differences in interaction architecture associated with polyploid frequency. Our work highlights the need for future research on polyploidy-driven shifts at both the community and the species levels. This will help to further explore the mechanisms behind these phenomena and understand how they might be influenced by global changes in pollinator availability and climate-driven range shifts that alter plant community composition.

## Data Availability

The original contributions presented in the study are included in the article/**Supplementary Material**. Further inquiries can be directed to the corresponding authors.
