# Supplementary material for: The role of plant polyploidy in the structure of plant-pollinator communities
Source: Front Plant Sci. 2026 Feb 10;17:1676445. doi: 10.3389/fpls.2026.1676445 (PMC12929425; doi:10.3389/fpls.2026.1676445)

Supplementary materials: The role of plant polyploidy in the structure of plant-pollinator communities

Ido Zylberberg[1], Keren Halabi[1], Noa Ecker[2], Nathália Susin Streher[3], Tal Pupko[2], Tia-Lynn Ashman[3], Itay Mayrose[1]

[1] School of Plant Sciences and Food Security, George S. Wise Faculty of Life Sciences, Tel Aviv University, Tel Aviv 69978, Israel.

[2] The Shmunis School of Biomedicine and Cancer Research, George S. Wise Faculty of Life Sciences, Tel Aviv University, Tel Aviv 69978, Israel.

[3] Department of Biological Sciences, University of Pittsburgh, Pittsburgh, PA 15260, USA.

* To whom correspondence should be addressed:

Itay Mayrose, Tel: +972-3-6407212

E-mail: itaymay@tauex.tau.ac.il

Tia-Lynn Ashman, Tel: +412-624-0985

E-mail: tia1@pitt.edu

Supplementary Notes

Note S1: Flower restrictiveness data collection

Flower restrictiveness data were compiled for species with assigned ploidy level, totaling 1,448 plant species. Each species was characterized according to the degree to which floral morphology restricts pollinator access to reproductive organs or rewards, broadly following the functional definition used in Burns et al. (2019). Thus, flower restrictiveness as defined here broadly reflects the potential for pollinators access to floral rewards. We classified flowers using a four-level system based on overall floral form and accessibility, as detailed in the table below:

Restriction level	Description	Examples of floral morphology included
Unrestrictive	Free access to floral resources; no morphological barriers.	open, exposed reproductive organs; grass/grass-like, inconspicuous, brush, free petals.
Low restriction	Some structure guiding access, but still accessible to many visitors.	capitulum, campanulate (shallow); open funnelform (slightly fused petals, exposed reproductive organs); petals erect/pedant somewhat enclosing, narrow corolla opening without tube.
Moderate restriction	Access requires manipulation or moderate morphological/behavioral adaptations.	Papilionaceous flowers; short tube; wide opening tube, campanulate (deep/narrow); narrow funnelform (used petals, exposed reproductive organs); labellum triggering.
High restriction	Highly specialized access, requiring specific morphology or behavior.	long tube; narrow tube; nectar spur; poricidal anthers; gynostegium; highly modified labellum; trap mechanism; long beaked galea.

Flower restrictiveness information was obtained primarily by scanning iNaturalist images (www.inaturalist.org; accessed September 2025) and cross-referencing species descriptions available in curated online resources (e.g., eFloras: http://www.efloras.org/; GBIF:

https://www.gbif.org/; World Flora Online: https://www.worldfloraonline.org/). When not available, we searched for information in peer-reviewed literature and published datasets (e.g., Bennett et al., 2018; Lanuza et al., 2023). Supplementary File S3 provides the full restrictiveness classification for each species, including the assigned category, source information, and a brief morphological justification that matches the table above.

Note S2: Path analysis for modeling the effect of polyploid frequency on network structure – statistical verification.

To assess the robustness of the path analysis, we explored several variations to the initial diagram described in the main text. First, since our base model only accounted for the effect of precipitation on network structure, we examined an alternative diagram where BIO15 (Precipitation seasonality) was replaced with BIO10 (mean temperature of warmest quarter). The results suggested that networks collected from areas with higher temperature are significantly less connected and robust. The effect of %PP on network indices remained similar to those reported in the main text. Second, we tested whether inclusion of both BIO10 and BIO15 could reveal causal relationships that were not accounted for when considering each class of environmental factor individually (Fig. S4). The inclusion of both BIO10 and BIO15 in the diagram had minor effect on the paths leading form the three plant traits (%PP, %SC, and %Restrictive) to the four network indices (Fig. S5). Furthermore, the inclusion of BIO10 to the baseline diagram had little contribution to the explanatory power of the model, as evident by the negligible increase in R^2 values compared to those described in the main text (Fig. 3 and Fig. S5). Third, we examined the separate effects of the frequencies of plants with restrictive flowers and self-compatible plants by fitting the structural equational models while either excluding the %Restrictive factor or %SC factor from the diagram (Fig. S6). This analysis revealed that %SC contributes somewhat more to the overall explanatory power of the model compared to %Restrictive, as evident from the consistently higher R^2 values in the %Restrictive-excluded models compared to %SC-excluded ones. The effect on the association of %PP with the four network indices was similar in both cases.

Note S3: Extinction simulations for modeling the effect of polyploidy on robustness

To examine the explicit effect of polyploid presence within communities on network resilience, we performed extinction simulations using the stochastic coextinction model (SCM) of Vieira & Almeida-Neto (2015). The SCM describes both deterministic and stochastic aspects of coextinctions and can simulate secondary coextinction cascades triggered by primary extinction events. We considered a species extinct when it lost all its interactions in the network.

Here, we used SCM to simulate a complete extinction process by repeatedly selecting a single plant species for primary extinction and triggering a SCM cascade until no interactions remain in the network. Three sets of simulations were generated, each consisting of 100 extinction simulations per network. In the first set, plant species were selected randomly for primary extinction until no interactions remained in the network (termed hereafter 'random extinction'). In the second set, polyploid species were initially sampled for primary extinction, followed by diploids and then plant species with ploidy unknown ploidy (termed hereafter 'polyploids-first extinction'). In the third set, diploid species were initially sampled for primary extinction, followed by polyploids and then plant species with unknown ploidy (termed hereafter 'diploids-first extinction'). Notably, in networks with only few species with available ploidy classification, the constrains on the sequence of species selected for primary extinction were too stringent in both the 'polyploids-first extinction' and 'diploids-first extinction' scenarios, leading to highly similar simulation outcomes. To mitigate this issue, we limited this analysis to networks containing a minimum of five diploid plants and five polyploid plants, resulting in a dataset of 121 networks.

For each extinction simulation, we computed the robustness index as the area under the extinction curve (Burgos et al. 2007). The robustness score of a network was then computed as the average robustness value across 100 simulations of each scenario. The distributions of mean robustness computed across simulations of each network for the three different scenarios are shown in Fig. S6. The observed distributions were quite similar, with slight left skewness in the "polyploids-first extinction" scenario and slight right skewness for the "diploids-first extinction" scenario. A less variable distribution was obtained for the "random extinction" scenario, possibly because the robustness values across these simulations were

less constrained in their selection of species for primary extinction, leading to more balanced mean robustness values per network.

We applied a Wilcoxon test to compare the robustness values computed based on "polyploids-first extinction" and "diploids-first extinction" scenarios. The results consistently showed that the extinction order of plants based on their ploidy level has no significant effect on network robustness (test statistic of 2041 with p-value = 0.7), further indicating that the presence of polyploids has a nonsignificant effect on community robustness, in accordance with our previous analyses. Overall, the results suggest that the effect of polyploid frequency on community robustness is indirect, and as such may be too subtle to be captured via a direct analysis that accounts only for polyploidy while ignoring other community traits.

Supplementary References

Bennett JM, Steets JA, Durka W, Vamosi JC, Arceo-Gómez G, Burd M, Burkle LA, Ellis AG, Freitas L, Li J, *et al.* 2018. Glopl, a global data base on pollen limitation of plant reproduction. *Sci. Data* **5**: 1–9.

Bivand R. **2022**. R Packages for Analyzing Spatial Data: A Comparative Case Study with Areal Data.”. **54**: 488–518.

Lanuza JB, Rader R, Stavert J, Kendall LK, Saunders ME, Bartomeus I. **2023**. Covariation among reproductive traits in flowering plants shapes their interactions with pollinators. *Functional Ecology* **37**: 2072–2084.

Burgos E, Ceva H, Perazzo RPJ, Devoto M, Medan D, Zimmermann M, María Delbue A. **2007**. Why nestedness in mutualistic networks? *Journal of Theoretical Biology* **249**: 307–313.

Burns JH, Bennett JM, Li J, Xia J, Arceo-Gómez G, Burd M, Burkle LA, Durka W, Ellis AG, Freitas L, *et al.* 2019. Plant traits moderate pollen limitation of introduced and native plants: a phylogenetic meta-analysis of global scale. *New Phytologist* **223**: 2063–2075.

Kissling WD, Carl G. **2008**. Spatial autocorrelation and the selection of simultaneous autoregressive models. *Global Ecology and Biogeography* **17**: 59–71.

Venables WN, B. D. Ripley. **2002**. *Statistics complements to modern applied statistics with S Fourth edition.*

Vieira MC, Almeida-Neto M. **2015**. A simple stochastic model for complex coextinctions in mutualistic networks: robustness decreases with connectance. *Ecology Letters* **18**: 144–152.

Supplementary Tables

Table S1. Results of a univariate regression analysis on all indices, using spatial autoregressive models. For each network-based index, the correlation coefficient ($\boldsymbol{R}$), its standardized coefficient ($\boldsymbol{\beta}$), and its LRT-based p-value ($\boldsymbol{p}$) are shown for each examined predictor. Significant coefficients are bolded.

predictor	N networks	Connectance		Nestedness		Modularity		Robustness	
		R (β)	p	R (β)	p	R (β)	p	R (β)	p
%PP[a]	325	-0.014 (-1.106)	0.274	0.151 (1.442)	0.153	-0.147 (-0.643)	0.532	-0.012 (-1.211)	0.229
%SC[b]	101	-0.044 (0.387)	0.718	0.015 (1.765)	0.098	0.144 (0.492)	0.636	-0.044 (0.245)	0.813
%Restrictive[c]	316	-0.014 (-0.48)	0.632	0.077 (0.755)	0.451	0.1 (0.861)	0.394	0.135 (-0.137)	0.895
BIO4[d]	325	**-0.144 (-2.929)**	**0.002**	**-0.07 (-3.815)**	**0**	0.05 (1.617)	0.102	**-0.295 (-6.083)**	**0**
BIO10[e]	325	0.004 (0.265)	0.791	0.023 (-0.419)	0.676	0.109 (0.978)	0.33	-0.018 (-0.22)	0.826
BIO15[f]	325	**-0.188 (-3.14)**	**0.001**	**-0.148 (-4.006)**	**0**	0.027 (0.763)	0.444	**-0.323 (-5.615)**	**0**
BIO18[g]	325	0.075 (1.71)	0.086	**0.01 (2.999)**	**0.003**	-0.004 (-0.977)	0.33	**0.149 (4.522)**	**0**
Network size	325	**0.224 (3.46)**	**0.001**	**0.153 (2.608)**	**0.009**	**0.304 (4.624)**	**0**	**0.178 (2.549)**	**0.011**

[a]Frequency of polyploid species in the network; [b]Frequency of self-compatible species; [c]Frequency of species with restrictive floral morphology; [d]Temperature seasonality; [e]Mean temperature of warmest quarter; [f]Precipitation seasonality; [g]Precipitation of warmest quarter.

Supplementary Figures

Figure S1. The distribution of collected plant-pollinator visitation networks across the globe, in the networks used for the path analysis (n=325)

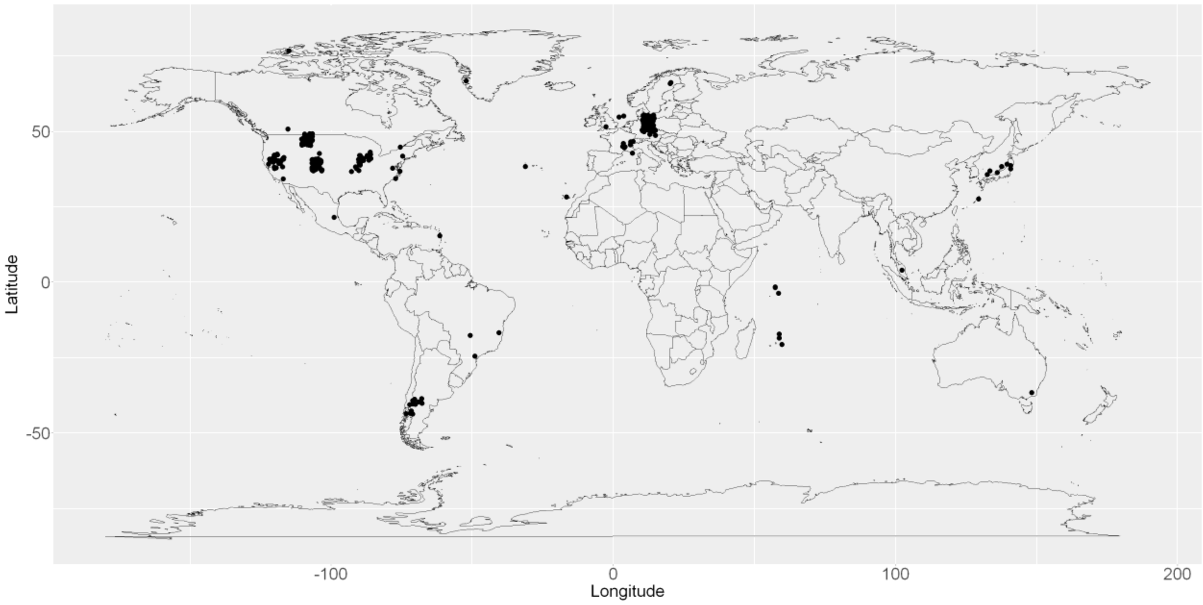

Figure S2. The distribution of plant trait data across the analyzed networks. The frequency distribution of polyploids (%PP), plants with flowers that restrict pollinator access (%Restrictive), and self-compatible plants (%SC) are shown across the 313 analyzed networks from the path analysis, in panels (a), (b), and (c), respectively. The distributions of missing data for ploidy, flower restrictiveness, and mating system classifications are shown in panels d-f, respectively.

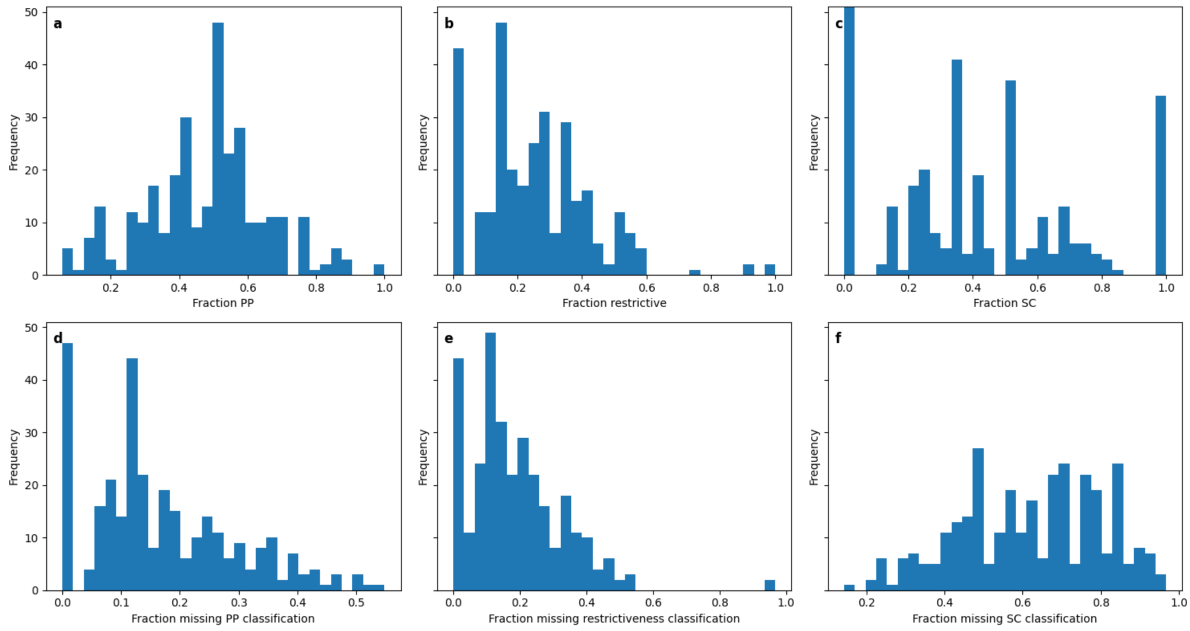

Figure S3: Path analysis using stricter filtering. Path diagrams of the four examined network indices used in the path analysis, when filtering for at least 6 plants classified for SC and restrictiveness. This filter is used in addition to the at least 6 plants and pollinators, and at least 6 plants classified with ploidy, constituting at least 33% of total plants as used in the main analysis presented in the main text. Full lines correspond to paths with significant contribution and dashed lines correspond to paths with nonsignificant contribution. Orange lines correspond to negative coefficients and blue to positive ones. The $\boldsymbol{R^2}$ is shown next to each network index. In all panels, the results of the $\boldsymbol{\chi^2}$ test for model adequacy were non-significant ($\boldsymbol{p = 0.153}$), indicating that the model is adequate.

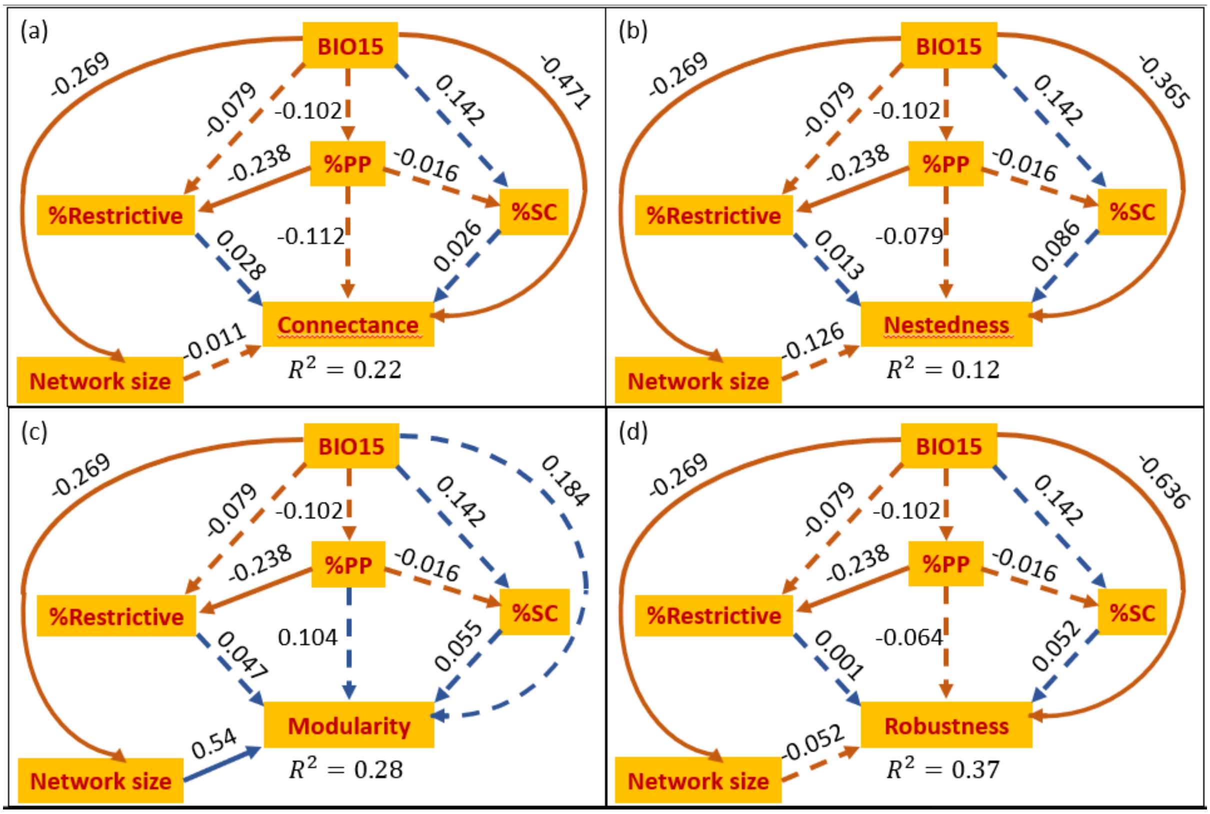
(a)
BIO15
%PP
%Restrictive
%SC
Connectance
Network size
-0.269
-0.471
-0.079
-0.102
0.142
-0.238
-0.016
-0.112
0.028
0.026
-0.011
$R^2 = 0.22$
(b)
BIO15
%PP
%Restrictive
%SC
Nestedness
Network size
-0.269
-0.365
-0.079
-0.102
0.142
-0.238
-0.016
-0.079
0.013
0.086
-0.126
$R^2 = 0.12$
(c)
BIO15
%PP
%Restrictive
%SC
Modularity
Network size
-0.269
0.184
-0.079
-0.102
0.142
-0.238
-0.016
0.104
0.047
0.055
0.54
$R^2 = 0.28$
(d)
BIO15
%PP
%Restrictive
%SC
Robustness
Network size
-0.269
-0.636
-0.079
-0.102
0.142
-0.238
-0.016
-0.064
0.001
0.052
-0.052
$R^2 = 0.37$

Figure S4: Path analysis using temperature (BIO10) as the environmental factor. Path diagrams of the four examined network indices used in the second set of path analysis, in which the environmental factor was BIO10: connectance (a), nestedness (b), modularity (c) and robustness (d). Full lines correspond to paths with significant contribution and dashed lines correspond to paths with nonsignificant contribution. Orange lines correspond to negative coefficients and blue to positive ones. The $\boldsymbol{R^2}$ is shown next to each network index. In all panels, the results of the χ^2 test for model adequacy were non-significant ($\boldsymbol{p} = \mathbf{0.159}$), indicating that the model is adequate.

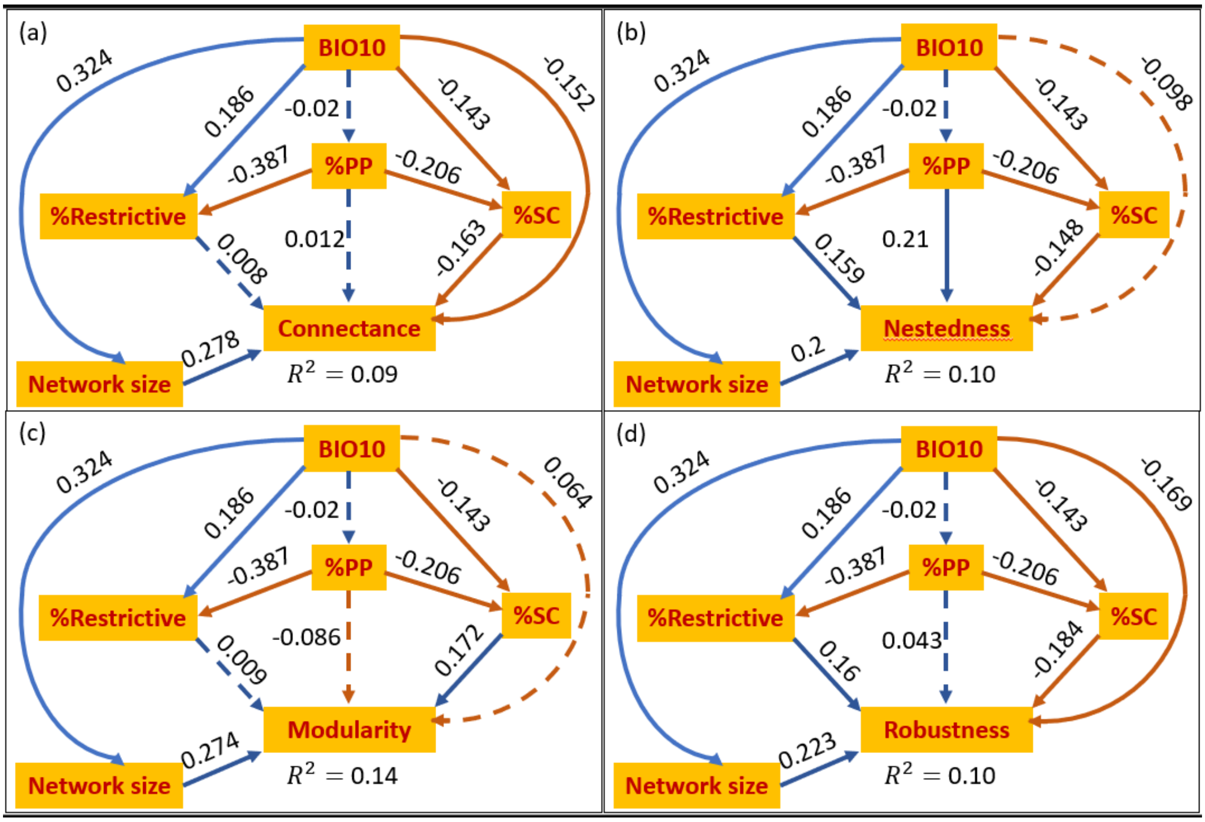

Figure S5: Path analysis using two environmental factors. Path Diagrams of the four examined network indices with both BIO10 and BIO15 included as environmental factors: connectance (a), nestedness (b), modularity (c) and robustness (d). Solid lines correspond to paths with significant contribution and dashed lines correspond to paths with nonsignificant contribution. Orange lines correspond to negative coefficients and blue to positive ones. The $\boldsymbol{R^2}$ is shown next to each network index. In all panels, the results of the $\boldsymbol{\chi^2}$ test for model adequacy were non-significant ($\boldsymbol{p = 0.268}$), indicating that the model is adequate.

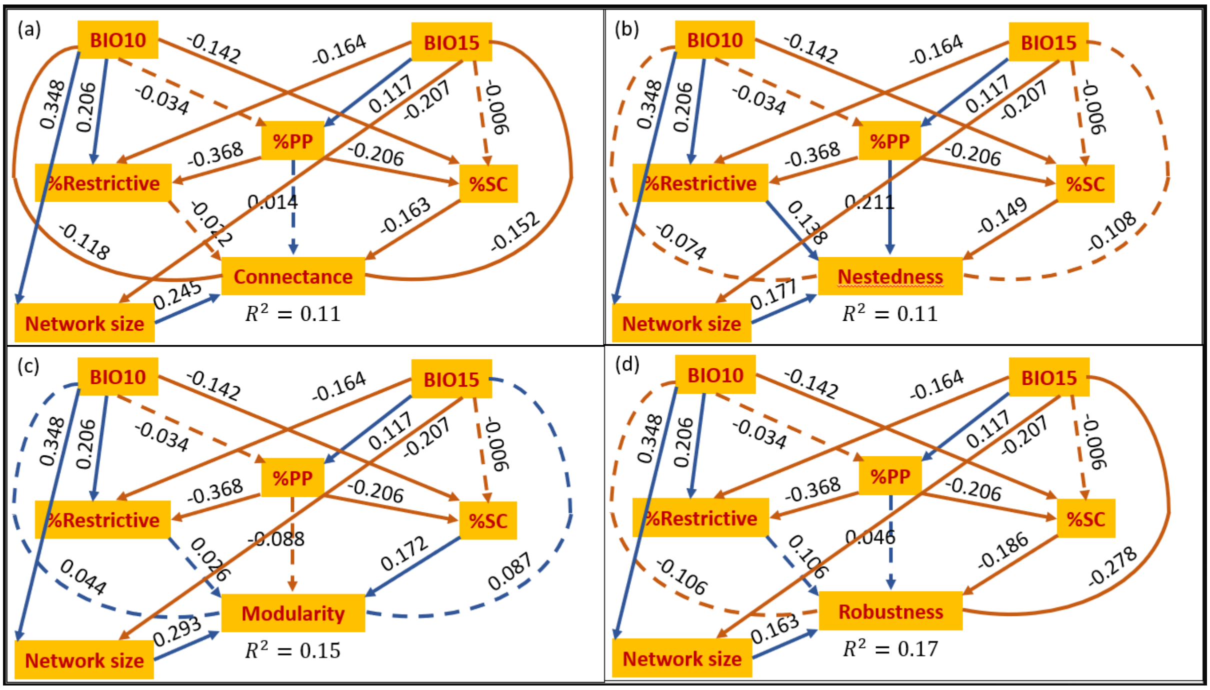

Figure S6: Path analysis with selective trait exclusion. Partial diagrams of the four examined network indices: connectance (a,b), nestedness (c,d), modularity (e,f) and robustness (g,h), with %Restrictive excluded (a,c,e,g) or %SC excluded (b,d,f,h). Solid lines correspond to paths with significant contribution and dashed lines correspond to paths with non-significant contribution. Orange lines correspond to negative coefficients and blue to positive ones. The $\boldsymbol{R^2}$ is shown next to each network index. In all panels, the results of the χ^2 test for model adequacy were non-significant ($\boldsymbol{p = 0.089}$ for exclusion of %Restrictive and $\boldsymbol{p = 0.07}$ for exclusion of %SC), indicating that the models are adequate.

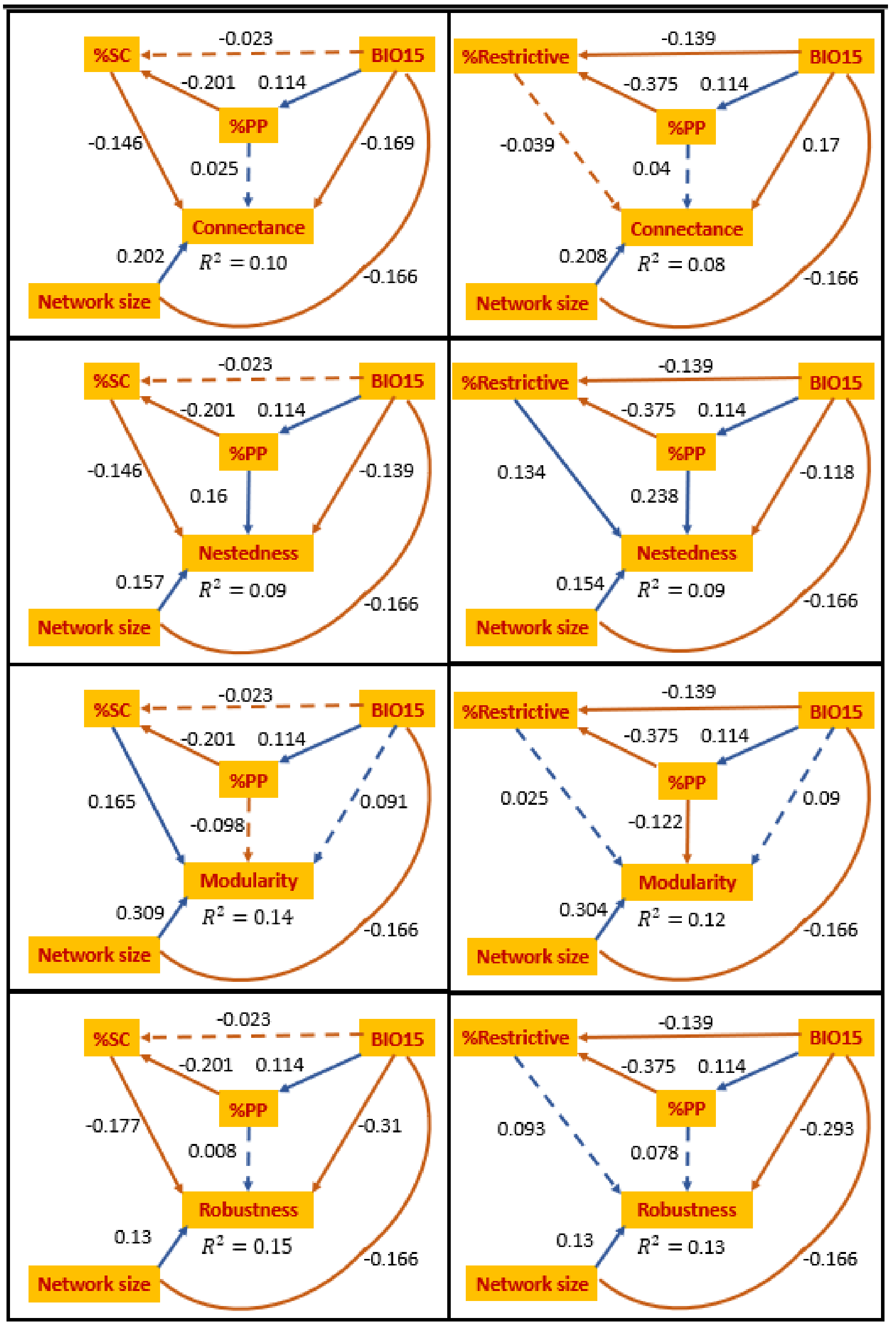

Figure S7. Distributions of robustness values under different extinction simulation scenarios. The distribution of mean robustness values across binarized networks is shown, as computed across extinction simulation scenarios with different primary extinction orders: random (grey), polyploids first (orange), and diploids first (blue). The analysis included 121 weighted networks that met the following criteria: at least 50% plant species with available ploidy classification, at least six pollinators, and at least 10 classified plant species (including a minimum of five polyploids and five diploids).

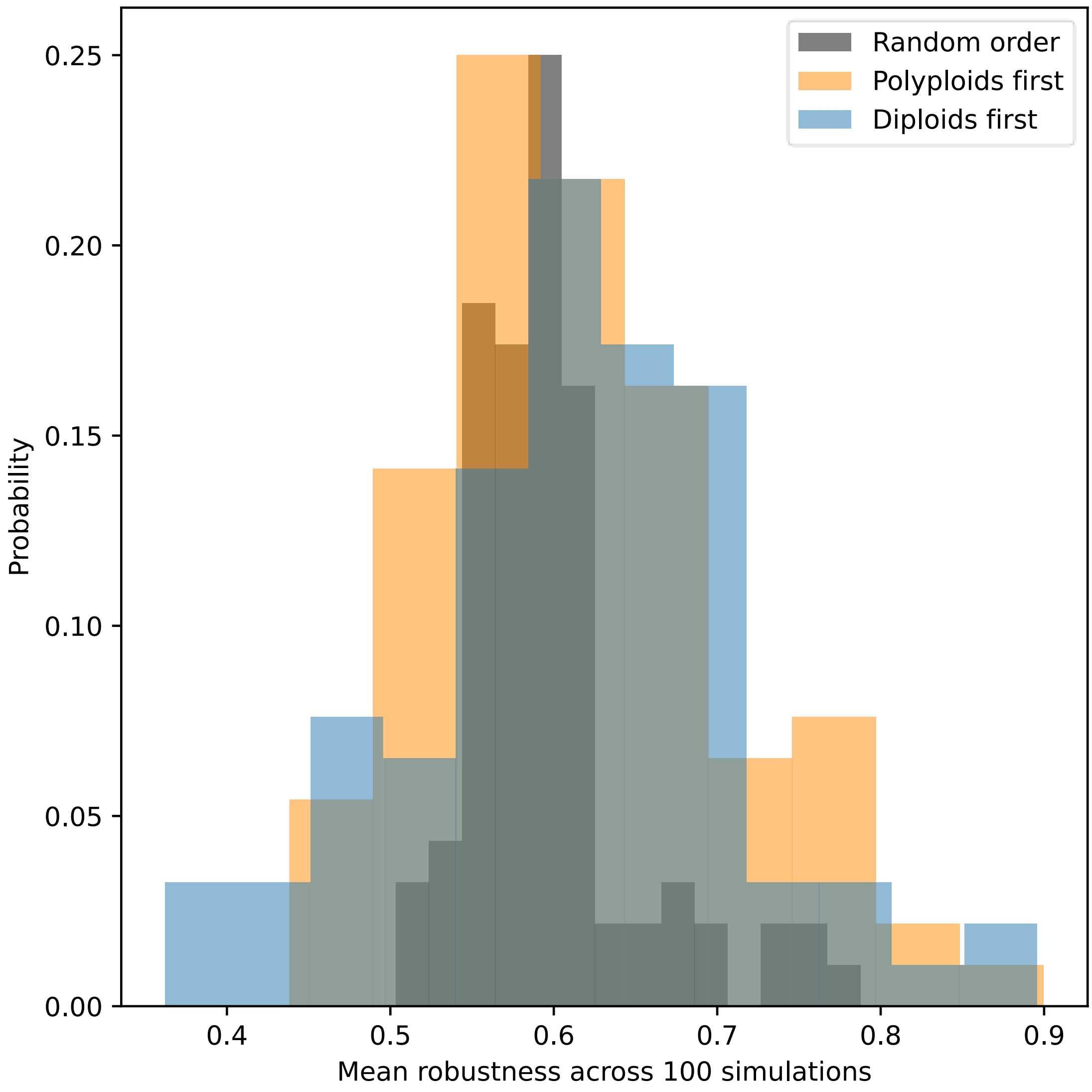

Supplement: Supplementary file 5 [file DataSheet5.pdf]
